# Effect of Feed Restriction on Performance and Postprandial Nutrient Metabolism in Pigs Co-Infected with *Mycoplasma hyopneumoniae* and Swine Influenza Virus

**DOI:** 10.1371/journal.pone.0104605

**Published:** 2014-08-07

**Authors:** Nathalie Le Floc'h, Céline Deblanc, Roland Cariolet, Anne V. Gautier-Bouchardon, Elodie Merlot, Gaëlle Simon

**Affiliations:** 1 INRA, UMR1348 PEGASE, Saint-Gilles, France; 2 Agrocampus Ouest, UMR1348 PEGASE, Rennes, France; 3 Anses, Ploufragan/Plouzané Laboratory, Swine Virology Immunology Unit, BP53, Ploufragan, France; 4 European University of Brittany, Rennes, France; 5 Anses, Ploufragan/Plouzané Laboratory, Production of Decontaminated Pigs and Testing, BP53, Ploufragan, France; 6 Anses, Ploufragan/Plouzané Laboratory, Mycoplasmology Bacteriology Unit, BP53, Ploufragan, France; INIA, Spain

## Abstract

As nutritional status and inflammation are strongly connected, feeding and nutritional strategies could be effective to improve the ability of pigs to cope with disease. The aims of this study were to investigate the impact of a feed restriction on the ability of pigs to resist and be tolerant to a coinfection with *Mycoplasma hyopneumoniae* (Mhp) and the European H1N1 swine influenza virus, and the consequences for nutrient metabolism, with a focus on amino acids. Two groups of specific pathogen-free pigs were inoculated with Mhp and H1N1 21 days apart. One group was fed *ad libitum*, the other group was subjected to a two-week 40% feed restriction starting one week before H1N1 infection. The two respective mock control groups were included. Three days post-H1N1 infection, 200 g of feed was given to pigs previously fasted overnight and serial blood samples were taken over 4 hours to measure plasma nutrient concentrations. Throughout the study, clinical signs were observed and pathogens were detected in nasal swabs and lung tissues. Feed-restricted pigs presented shorter hyperthermia and a positive mean weight gain over the 3 days post-H1N1 infection whereas animals fed *ad libitum* lost weight. Both infection and feed restriction reduced postprandial glucose concentrations, indicating changes in glucose metabolism. Post-prandial plasma concentrations of the essential amino acids histidine, arginine and threonine were lower in co-infected pigs suggesting a greater use of those amino acids for metabolic purposes associated with the immune response. Altogether, these results indicate that modifying feeding practices could help to prepare animals to overcome an influenza infection. Connections with metabolism changes are discussed.

## Introduction

In pigs, infectious diseases and inflammatory states induce metabolic alterations and changes in nutrient partitioning between tissues associated with growth (e.g. muscle) and those involved in body defence (liver, lymphoid organs, etc.) [1], [2]. Metabolism disruption during disease or inflammation has also been demonstrated and is well-known in humans [3]–[5]. Conversely, many nutrients are directly or indirectly involved in the regulation of inflammatory and immune responses [6]–[8]. Particularly, amino acids (AA) are redistributed away from muscle protein synthesis towards tissues and cells involved in inflammatory and immune responses either to be used for the synthesis of specific compounds or to be degraded for use as energetic precursors [1]. Consequently, both utilization of dietary AA and intensity of AA mobilization may influence the ability of pigs to resist to an infectious agent and limit the detrimental consequences of the inflammation caused by the response to infection.

Infectious diseases and inflammatory states decrease feed consumption [9] and may induce a nutrient deficiency that can, in turn, reduce the ability of animals to mount an efficient immune response. Conversely, it has been shown that a feed restriction can have a beneficial effect on animal health [10], [11]. Indeed, in mice, a 40% feed restriction decreased the acute release of pro-inflammatory cytokines and induction of inducible nitric oxide synthase expression in response to an LPS challenge, thereby avoiding extreme inflammatory responses which could damage organs [10]. In growing pigs, caloric restriction modified the expression of immune genes [12] suggesting that a short-term feed restriction may modify the immune response. However, the beneficial effect of feed restriction on pig health remains to be established for diseases other than digestive diseases [11]. The feed cost represents more than 60% of the pig productive cost. Moreover, after a period of feed restriction, pigs usually exhibite a compensatory growth that limits the impact of a transient limitation of feed intake [13]. Thus, strategies based on a moderate and transient feed restriction deserve to be considered to reduce the impact of health problems and to maintain the productivity of farm animals without increasing feed cost.

In modern pig production, respiratory diseases are common and one responsible pathogen is the swine influenza virus (SIV) [14]. Three SIV subtypes (H1N1, H1N2 and H3N2) are simultaneously circulating among pigs worldwide, but genetic lineages may vary within each subtype depending on the region (i.e. Europe, Asia and North America) [15]. Swine influenza is characterized by hyperthermia, loss of appetite, lethargy, respiratory problems and coughing, similarly as human flu. The severity of the disease is highly variable and influenced by many factors such as the virus strain, the age and immune status of the infected pig, the housing environment, and the presence of other respiratory pathogens [16]–[19]. The infection is restricted to the respiratory tract, without viremia [20]. It has an impact on animal health and welfare but also generates important economic losses because of the decline in animal performance and the medication costs in the case of complications. Recently, the European avian-like swine H1N1 virus subtype has been identified in pig herds as a major pathogen involved in the Porcine Respiratory Disease Complex, together with *Mycoplasma hyopneumoniae* (Mhp) [21], another respiratory pathogen widespread in French farms. Alone, Mhp induces chronic pneumonia with dry, non productive cough beginning 7 to 14 days after infection. Under experimental conditions, the diseases induced by intra-tracheal inoculation of Mhp or H1N1 mimicked respiratory outbreaks observed in the herds of origin [17], [22] and it was shown that pre-infection of pigs with Mhp increased the severity of clinical symptoms induced by H1N1 [17], inducing a strong inflammatory response [18].

The aim of the present study was to evaluate the impact of 1) a Mhp/H1N1 co-infection on nutrient metabolism and 2) a 40% feed restriction on the ability of Mhp/H1N1 co-infected pigs to resist and to cope with the metabolic consequences of inflammation caused by infection (refered as tolerance) [23]. Resistance and tolerance to the infection were assessed by measuring pathogen excretions, clinical signs, and changes in nutrient metabolism, with a special focus on AA. A meal standardized for size, duration and composition was used to study post-prandial AA utilization and to identify which AA exhibited modified metabolism.

## Materials and Methods

### Pathogens and animals

The two pathogens, *M. hyopneumoniae* strain 116 and the avian-like swine H1N1 virus strain A/Sw/Cotes d'Armor/0231/06, were isolated from French farms. Strain isolations, as well as preparation and titration of inocula were performed as previously described [17].

Twenty-four specific pathogen-free pure-bred 6-week-old Large White pigs were randomly assigned to this study, taking into account litter origin, weight and sex. Young males were castrated and had similar metabolism and growth rate to those of females. These animals were obtained from the experimental pig herd of the French Agency for Food, Environmental and Occupational Health and Safety (Anses) at Ploufragan. These animals were free from Mhp and SIV at the beginning of the study.

### Experimental design and sample collection

Experiments were performed in the Anses facilities in accordance with the animal welfare experimentation recommendations drawn up by the *Directions Départementales de la Protection des Populations des Côtes d'Armor* (Anses registration number B-22-745-1), under the responsibility of G. Simon (authorization number 22–26). This animal experiment protocol was approved by the French national ethics committee ComEth Anses/ENVA/UPEC (approval n°15/02/11-7). Infected and Control pigs were housed in separate rooms. Very strict biosecurity measures, i.e air filtration system and airlocks for each room, room-specific clothes and compulsory showering before and after visiting the pigs were implemented to avoid undesirable contamination of the pigs (BSL3).

Two groups of 8 pigs were intra-tracheally inoculated on two consecutive days with 5 ml of 10^8^ Colour Changing Unit (CCU)/ml of Mhp 116 (at Day (D) 0 and D1) and, 3 weeks later (at D21), with 10^5^ Embryonic Lethal Dose (ELD_50_) of H1N1 in a total of 5 ml (MH1N1 groups). Simultaneously, two control groups of 4 pigs each received intra-tracheally pathogen culture media, i.e. 5 ml of Friis broth medium at D0 and D1, and 5 ml of allantoic fluid at D21 (C groups). All pigs were transferred to individual pens one week before H1N1 infection (D15). Drinking water was provided *ad libitum* to all groups throughout the study. One infected group and one control group were fed *ad libitum* (MH1N1-AL and C-AL groups) whereas the other two groups were subjected to a 40% feed restriction (MH1N1-FR and C-FR groups) from D15 until the end of the experiment (Table 1). Pigs were fed a standard starter diet until D14 then a standard growing diet until the end of the experiment. These diets were formulated to cover the nutritional requirements of pigs (see Table S1). The level of 40% feed restriction was determined in a preliminary experiment as the highest level of restriction that did not induce body weight loss (unpublished data).

**Table 1:** Experimental design.

Groups	Nb. of pigs	Intra-tracheal inoculations		Feed from Day 15 to Day 28
		Day 0+Day 1	Day 21 (0 DPI H1N1)	
MH1N1-AL	8	*Mycoplasma hyopneumoniae*	H1N1	*ad libitum*
C-AL	4	Friis broth medium	allantoïc fluid	*ad libitum*
MH1N1-FR	8	*Mycoplasma hyopneumoniae*	H1N1	40% feed restriction
C-FR	4	Friis broth medium	allantoïc fluid	40% feed restriction

After an overnight fasting, the pigs were surgically fitted with a jugular catheter at D14 (control groups) or at D16 (infected groups) under general anaesthesia (premedication with ketamine, 400 mg/pig IM, and anaesthesia with propofol, 40 mg/pig IV).

Three days post-infection (DPI) with H1N1 (i.e. at D24), after an overnight fasting, 200 g of feed were given to all animals. The meal size was calculated to ensure that all pigs ate their meal in less than 10 minutes. Blood samples (4 ml) were sampled in heparinized tubes just before the distribution of the test meal, then every 15 min during the first 2 hours and every 30 min during the next 2 hours, for measurement of plasma postprandial glucose, urea and AA concentrations.

Additional blood samples were taken in heparinized tubes 4 days before Mhp inoculation (D0), on D17 (i.e., 4 days before H1N1 inoculation) and on D28 for determination of total IgG levels. All plasmas were separated by centrifugation and stored at −20°C until use.

Pigs were examined daily for clinical signs (rectal temperature, coughing, respiratory rates, and feed consumption) throughout the study and weighed on days 0, 7, 16, 21, 24 and 28. Nasal swabs were taken on days 23, 24 and 28 (i.e., 2, 3 and 7 DPI with H1N1) for determination of viral excretion. After collection, swabs were suspended in 2 ml of Eagle's minimum essential medium (EMEM, LONZA, Levallois-Perret, France) containing penicillin (100 UI/ml) and streptomycin (100 µg/ml) (Sigma, Saint-Quentin Fallavier, France), vigorously vortexed and then the supernatants were stored at −70°C. One week post-H1N1 infection (on D28), the pigs were euthanized by intravenous injection of sodium pentobarbital followed by exsanguination. Post-mortem examinations were immediately carried out and macroscopic pulmonary lesions were scored visually as previously described [24]. A sample of each (apical, cardiac and diaphragmatic) left lobe of the lungs was collected for detection of Mhp and SIV and stored at −70°C until use. A sample of the left diaphragmatic lobe was also fixed in 10% neutral buffered formalin for histopathological examination, as previously described [17]. The severities of hyperplasia of the bronchial epithelium, hyperplasia of the bronchiolar-associated lymphoid tissue, interstitial pneumonia and bronchiolitis were scored between 0 ( = no lesion) and 4 (lesion very marked) in order to obtain a total score of 16.

### Detection and quantification of pathogens

For Mhp detection and quantification, DNA was extracted from 25 mg of lung samples with DNeasy Blood and Tissue kit (Qiagen, Courtaboeuf, France) and analyzed by specific PCR assay as previously described [22].

H1N1 virus genome was detected in nasal swab supernatants and lung samples by matrix (M) gene real-time RT-PCR. RNA was extracted from 200 µl of nasal swab supernatants and from 30 mg of lung samples with the RNeasy Mini kit (Qiagen, Courtaboeuf, France) and tested using the TaqVet Influenza A, INFAP-Swine kit (LSI, Lissieux, France) [25] according to the manufacturer's instructions. M gene positive nasal swab supernatants were then titrated in MDCK cells as previously described [17] to determine the H1N1 particle content.

### Total IgG assay

Total IgG concentration was determined by quantitative sandwich ELISA, using goat antibodies directed against the swine IgG Fc fragment and a pig immunoglobulin reference serum for the standard curve (A100-104A, A100-104P and RS10-107 respectively, Bethyl, Interchim, Montluçon, France). The colorimetric reaction following the addition of 3,3′,5,5′-Tetramethylbenzidine (TMB) substrate (Sigma–Aldrich, Saint Quentin Fallavier, France) was halted by adding H_2_SO_4_ 2.5N and absorbance was read with a microplate reader at a test wavelength of 450 nm.

### Plasma postprandial nutrient measurement

Plasma concentrations of glucose, urea and AA were determined from blood collected during the test meal. Concentrations of glucose and urea were determined using the Glucose RTU kit (Biomérieux, Marcy l'Etoile, France) and UREA UV 250 kit (Biomérieux, Marcy l'Etoile, France), respectively, with a Konelab 20i analyzer (ThermoFisher Scientific). The intra-assay CV was less than 5%. Plasma AA concentrations were determined by an ultra performance liquid chromatography (UPLC) system (Waters Acquity Ultra Performance LC, Waters, Milford, MA, USA) coupled to two detectors (Acquity Tunable UV detector and Mass SQD detector; Waters, Milford, MA, USA) after derivatization of samples using the AccQ·Tag Ultra method (MassTrak AAA; Waters, Milford, MA, USA). Norvaline (Sigma-Aldrich, Saint Quentin Fallavier, France) was used as an internal standard. This method allowed separation and identification of 33 AA in plasma, 21 of which were analysed statistically: 20 are proteinogenic AA (cysteine was not analysed because unstable during sample conservation) plus citrulline because of its involvement in arginine metabolism. The other AA were either detected at very low level in the plasma, or not directly related to dietary AA.

For each individual kinetic of AA, glucose and urea, the area under the curve (AUC) was calculated as the sum of trapeze areas. For that purpose, it is assumed that the shape of nutrient change between two consecutive sampling times was linear and the area of each consecutive trapeze from x-axis was calculated.

### Statistical analyses

The clinical data, virus titrations and Mhp DNA quantifications obtained in the different groups were compared by Kruskal-Wallis test using Systat 9 software. Plasma IgG and postprandial nutrient concentrations were subjected to a variance analysis, using the MIXED procedure of SAS (SAS Inst. Inc., Cary, NC). Plasma total IgG concentrations were analyzed using the repeated statement and taking into account the day of sampling, feed restriction (FR), infection (I) and the corresponding interactions. Because of non-normal distribution and non- homogenate variances for glucose, urea and AA plasma concentrations due to the major effect of time, statistical analyses of these data was performed separately at each sampling time and data were log-transformed to be normalized when necessary. For nutrient concentrations and AUC, the model included the effects of feed restriction (FR), infection (I) and the corresponding interactions. For simplification, AA average concentrations measured at basal state, 60 and 240 min after the meal are presented as Supporting information (Tables S2, S3 and S4), whereas AUC values for each AA and the postprandial kinetics of glucose, urea, threonine and arginine were presented and commented in the Result section. The results are expressed as least square means calculated for each time and each experimental treatment.

For all statistical analyses, probabilities less than 0.05 were considered significant. Probability values for the effect of infection and feed restriction comprised between 0.05 and 0.10 were considered as a trend.

## Results

### Clinical disease

All pigs inoculated with Mhp were coughing 2 weeks later, demonstrating they were infected (data not shown). The week before H1N1inoculation, the average frequencies of coughs per pig per 15 min were similar in MH1N1-FR and MH1N1-AL groups (0.3±0.3 and 0.6±0.3, respectively; P = 0.07). At 1 DPI H1N1, they were higher but still similar (1.4 and 1.3, respectively). No coughing was observed in the two control groups.

**Figure 1:**
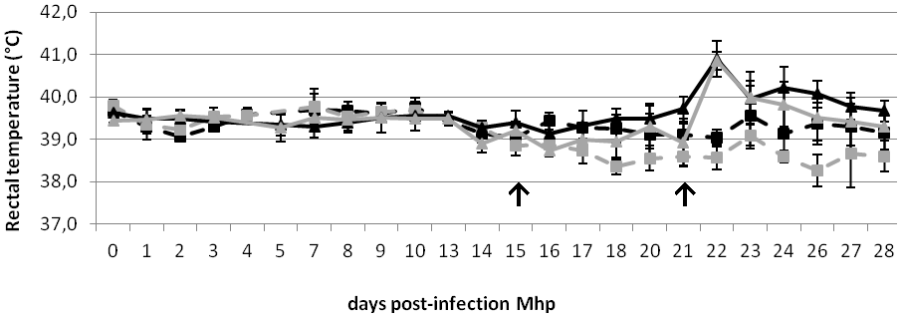
Mean rectal temperatures (°C) of pigs. Mean rectal temperatures in co-infected (MH1N1) and control (C) pigs fed *ad libitum* (AL) or feed restricted (FR) : C-AL (black dashed line), C-FR (gray dashed line), MH1N1-AL (black solid line) and MH1N1-FR groups (gray solid line), from the day of Mhp inoculation (day 0) until the end of the experiment. Arrows indicate the day of the beginning of the feed restriction (day 15) and the day of H1N1 inoculation (day 21).

During the first two weeks of the study, rectal temperatures were normal in all pigs, ranging from 39.2°C to 39.9°C (Figure 1). After feed restriction was applied, they decreased to 38.6±0.4°C and 39.0±0.4°C in C-FR and MH1N1-FR groups, respectively, leading to a significant difference (P<0.05) between FR and AL groups during the week before H1N1 inoculation. At 1 DPI H1N1, hyperthermia (temperature >40.0°C) was observed in both co-infected groups. A second peak of hyperthermia appeared in MH1N1-AL group between 3 and 5 DPI H1N1 and temperatures returned to normal at 6 DPI H1N1. In MH1N1-FR group, hyperthermia was of shorter duration, as temperature returned to normal from 2 DPI H1N1, leading to a significant difference between both co-infected groups at 5 DPI H1N1 (P = 0.02). No hyperthermia was observed in C groups and a significant difference due to the feed restriction was maintained between both C groups during the 4^th^ week of the assay (P<0.05).

The day after H1N1 inoculation, the mean respiratory rate increased similarly in both infected groups. It reached 65±23 and 62±16 breaths per min in MH1N1-AL and MH1N1-FR groups respectively, while it was maintained to 35 in both control groups.

**Figure 2:**
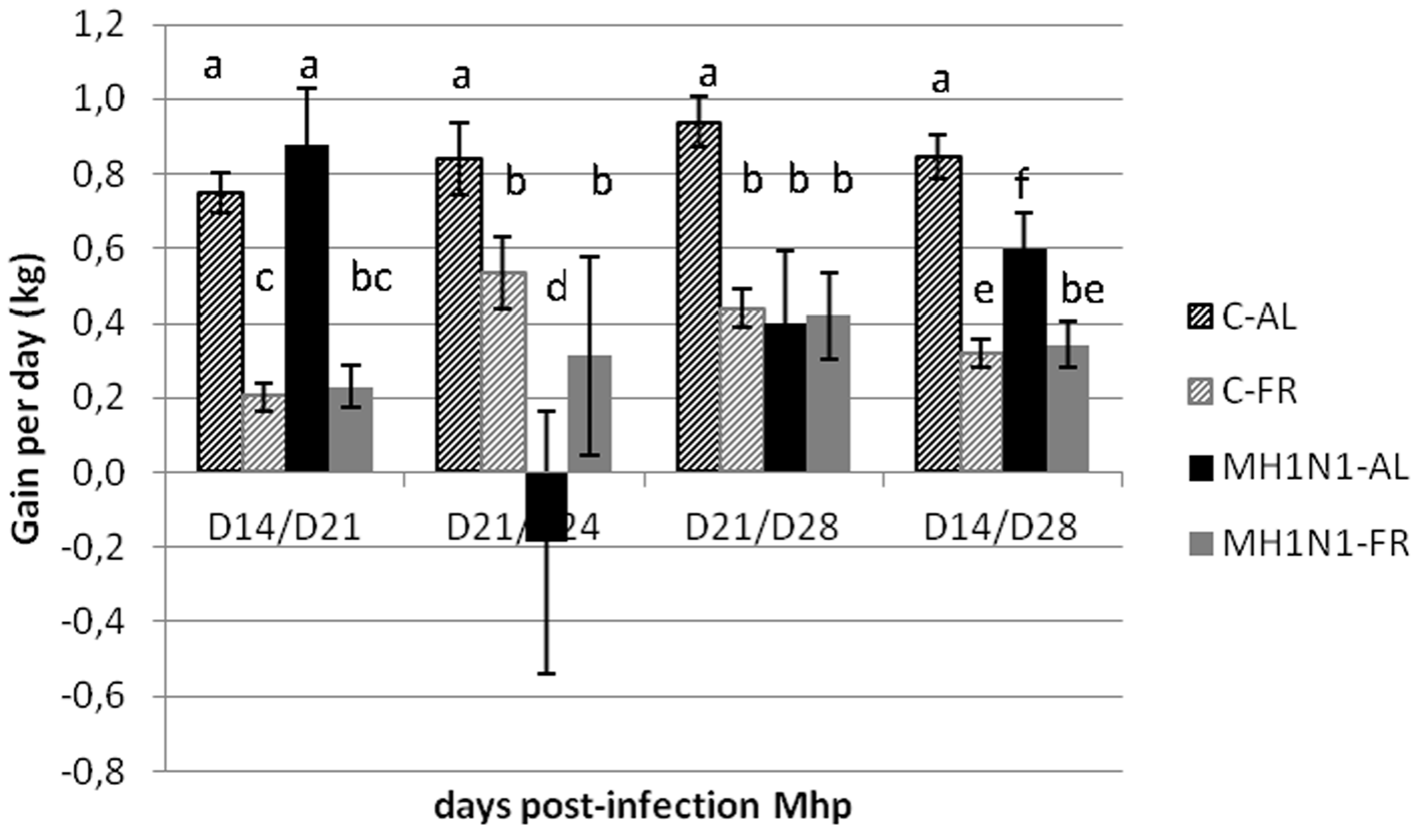
Mean daily weight gain (kg) of pigs after Mhp and H1N1 inoculations. Mean daily weight gains between Day (D) 14 and D21 (the week before H1N1 infection), between D21 and D24 (between 0 and 3 DPI H1N1), between D21 and D28 (between 0 and 7 DPI H1N1) and between D14 and D28, for control (C) or co-infected (MH1N1) groups fed *ad libitum* (AL) or feed restricted (FR): C-AL (hatched black), C-FR (hatched gray), MH1N1-AL (black) and MH1N1-FR (gray) groups. Mean daily weight gains with different letters are significantly different with P<0.05.

Viral infection led to a reduction in feed consumption in the MH1N1-AL group. It was strongly marked during the 3 first days, with pigs eating 30 g of feed per kg of body weight on average against 60 g before H1N1 infection (P = 0.001). This resulted in a reduction of weight and mean weight gain (MWG) as compared to other groups (P = 0.006) (Figure 2). In contrast, H1N1 infection had no impact on feed consumption in the MH1N1-FR group in which pigs were eating on average 30 g of feed per kg of body weight both before and after inoculation. In this group, MWG was identical to MWG measured in C-FR.

At necropsy, all co-infected pigs had macroscopic lesions of pneumonia (data not shown). They extended into apical, cardiac and diaphragmatic lobes with no difference in the mean score severity between MH1N1-AL and MH1N1-FR groups (17.4±3.3 and 16.5±3.5, respectively). In C-AL and C-FR groups, the scores were 0±0 and 2±2.8, respectively. Histological examinations showed that all co-infected animals developed bronchiolitis, broncho-interstitial and interstitial pneumonia (data not shown). The mean microscopic lesion scores were identical for both MH1N1-AL and MH1N1-FR groups (8.8±1.5 and 7.1±2.4, respectively). Microscopic lesions were not observed in pigs from the control groups.

### Detection and quantification of Mhp and H1N1 virus

Mhp genome was detected in all lung lobes from infected pigs without significant difference in DNA quantities between MH1N1-AL and MH1N1-FR groups (Table 2).

**Table 2:** Pathogen detections and quantifications in nasal swab supernatants and lung tissues of inoculated pigs.

Groups	Pig	H1N1 genome detection^a^/virus titration^b^						Mhp DNA quantification^c^		
		Nasal swabs			Lung tissues			Lung tissues		
		2 DPI	3 DPI	7 DPI	Apical lobe	Cardiac lobe	Diaphragmatic lobe	Apical lobe	Cardiac lobe	Diaphragmatic lobe
MH1N1-AL	1	+/ni	+/3.43	+/ni	+	+	+	19	101	19
	2	−/nt	+/3.56	+/ni	+	+	+	48	1851	4
	3	+/ni	+/3.4	+/1.63	+	+	−	544	1156	12
	4	−/nt	+/2.58	−/nt	+	+	−	164	8	2
	5	+/1.61	+/3.29	+/ni	+	−	+	16594	2496	2734
	6	+/2.71	+/2.71	+/ni	+	+	+	251	3397	9000
	7	+/1.92	+/2.57	+/ni	+	+	+	2814	1896	6576
	8	+/2.24	+/3.83	+/1.55	+	+	+	21	162	1
	Mean (SD)	2.12 (0.5)*	3.17 (0.5)*	1.59 (0.1)*	n.a.	n.a.	n.a.	2557 (5749)	1384 (1245)	2293 (3579)
MH1N1-FR	9	+/ni	+/3.35	−/nt	+	−	+	729	2100	535
	10	−/nt	+/1.63	+/ni	+	+	−	638	6	143
	11	+/ni	+/3.56	+/2.79	+	−	+	229	316	56
	12	+/3.39	+/3.71	+/ni	+	+	+	810	927	41
	13	−/nt	+/1.55	+/ni	+	+	−	658	49	976
	14	−/nt	+/ni	+/1.55	+	+	+	2705	2258	214
	15	+/2.75	+/3.61	+/1.56	+	+	+	2452	4131	9107
	16	+/1.75	+/3.61	+/ni	+	+	+	3449	4983	328
	Mean (SD)	2.63 (0.8) *	3.0(1)*	1.97 (0.7)*	n.a.	n.a.	n.a.	1459 (1212)	1846 (1894)	1425 (3119)

a Qualitative results of M gene real-time RT-PCR in nasal swab supernatants collected at 2, 3 and 7 DPI H1N1 and in lung tissues collected at necropsy (+ and − : positive and negative genome detection).

b Results of virus titration in nasal swab supernatants. Virus titers are expressed in log TCID50/ml (ni: not isolated; nt: not tested; *: averages calculated with values obtained from successful titrations).

c Results of Mhp quantification (expressed in pg DNA/25 mg organ) in lungs at necropsy. SD: standard deviation. N.a.: not applicable.

SIV RNA detection in nasal swabs showed that 6/8 pigs in the MH1N1-AL group and 5/8 pigs in the MH1N1-FR group shed H1N1 virus at 2 DPI H1N1, although the isolation and titration of viral particles was successful in only 4/8 and 3/8 animals, respectively (Table 2). At 3 DPI H1N1, all infected pigs were shedding the virus, with similar titers in both groups. At the end of the experiment (7 DPI H1N1), viral genome was detected in 7/8 animals in each infected group, and spread in the upper and lower lobes since the diaphragmatic lobes of 6/8 animals in both co-infected groups were detected positive.

### Plasma IgG concentrations

Feed restriction had no effect on variation of the plasma IgG concentrations (P_FR_ and P_I×FR_>0.1) (Table 3). The concentrations of IgG varied with time in MH1N1 pigs, but not in C pigs (P_I×FR_<0.01). Whatever the feeding level, the IgG concentrations in MH1N1 pigs were similar to those in C pigs before and 17 days after Mhp inoculation (P>0.1) but higher at 7 DPI H1N1 (P<0.001).

**Table 3:** Plasma total IgG concentrations (g/L) during the time course of the experiment in control (C) and co-infected (MH1N1) pigs.

	Day			SEM	P-value		
Group	D-4	D17	D28		I	T	IxT
C (-AL+-FR)	2.61^a^	1.75^a^	2.79^a^	0.87	<0.01	<0.001	<0.01
MH1N1 (-AL+-FR)	2.71^a^	2.23^a^	8.13^b^				

Standard errors of the mean (SEM) of control (C) and co-infected (MH1N1) conditions are presented with *ad libitum* (AL) and feed restricted (FR) pigs pooled together (C-AL+C-FR vs. MH1N1-AL+MH1N1-FR) because the effect of the feeding level (-AL or -FR) and its interactions with the effects of infection (I) and time (T) were not significant.

a,b : concentrations with different letters are significantly different with P<0.001.

### Postprandial profiles of plasma glucose, urea and amino acids

**Figure 3:**
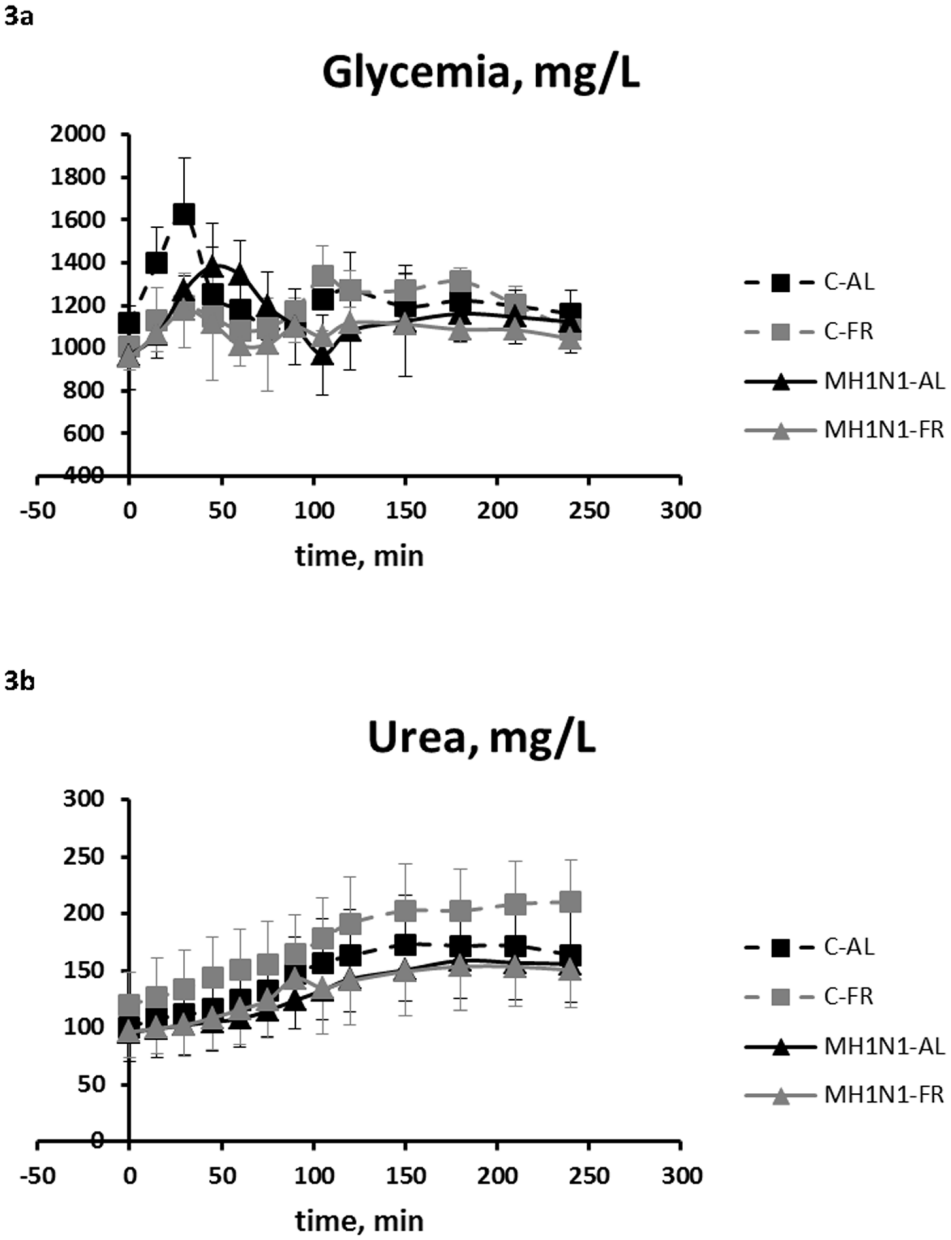
Postprandial kinetics of plasma glucose (3a) and urea (3b) concentrations (mg/L) measured in pigs. Average plasma concentrations were measured after an overnight fasting and then during the 4ïc fluid administration in co-infected (MH1N1) and control (C) pigs fed *ad libitum* (AL) or feed restricted (FR): C-AL (black dashed line), C-FR (gray dashed line), MH1N1-AL (black solid line) and MH1N1-FR groups (gray solid line). Values are means +/− SD. P values for glucose: P_I_<0.05 at fasted basal state, 15 and 30 min after the meal, then from 105 to 210 min; P_FR_<0.05 from 15 to 60 min after the meal. P values for urea: P_I_<0.01 from 60 to 240 min.

At 3 DPI H1N1 and after overnight fasting, 200 g of feed were given to all animals and plasma was regularly sampled during 4 hours. Two and 3 pigs from MH1N1-AL and MH1N1-FR, respectively, were not included because of a non-fonctionnal catheter. Co-infected pigs had lower glycemia at fasted basal state, 15 and 30 min after the meal, then from 105 to 210 min (P_I_<0.05) (Figure 3a). The peak of glucose was the highest in C-AL pigs around 30 min (P_I×FR_ = 0.03) whereas at 60 min glycemia was greater in MH1N1-AL pigs than in the three other groups (P_I×FR_ = 0.05). As a consequence, glucose AUC was also lower in MH1N1 pigs (P_I_ = 0.015). Feed restriction did not affect glucose AUC but modified the profile of glycemia that was lower in FR pigs than in AL pigs from 15 to 60 min after the meal (P_FR_<0.05).

Plasma urea increased linearly before reaching a plateau value at 150 min (Figure 3b). The effect of feed restriction and the interaction I×FR were not significant at any time. Urea AUC tended to be lower in MH1N1 pigs than in C pigs (P_I_ = 0.06). From 60 to 240 min, plasma urea concentrations were lower (P_I_<0.05) or tended (0.05<P_I_<0.1) to be lower in MH1N1 than in C pigs.

**Figure 4:**
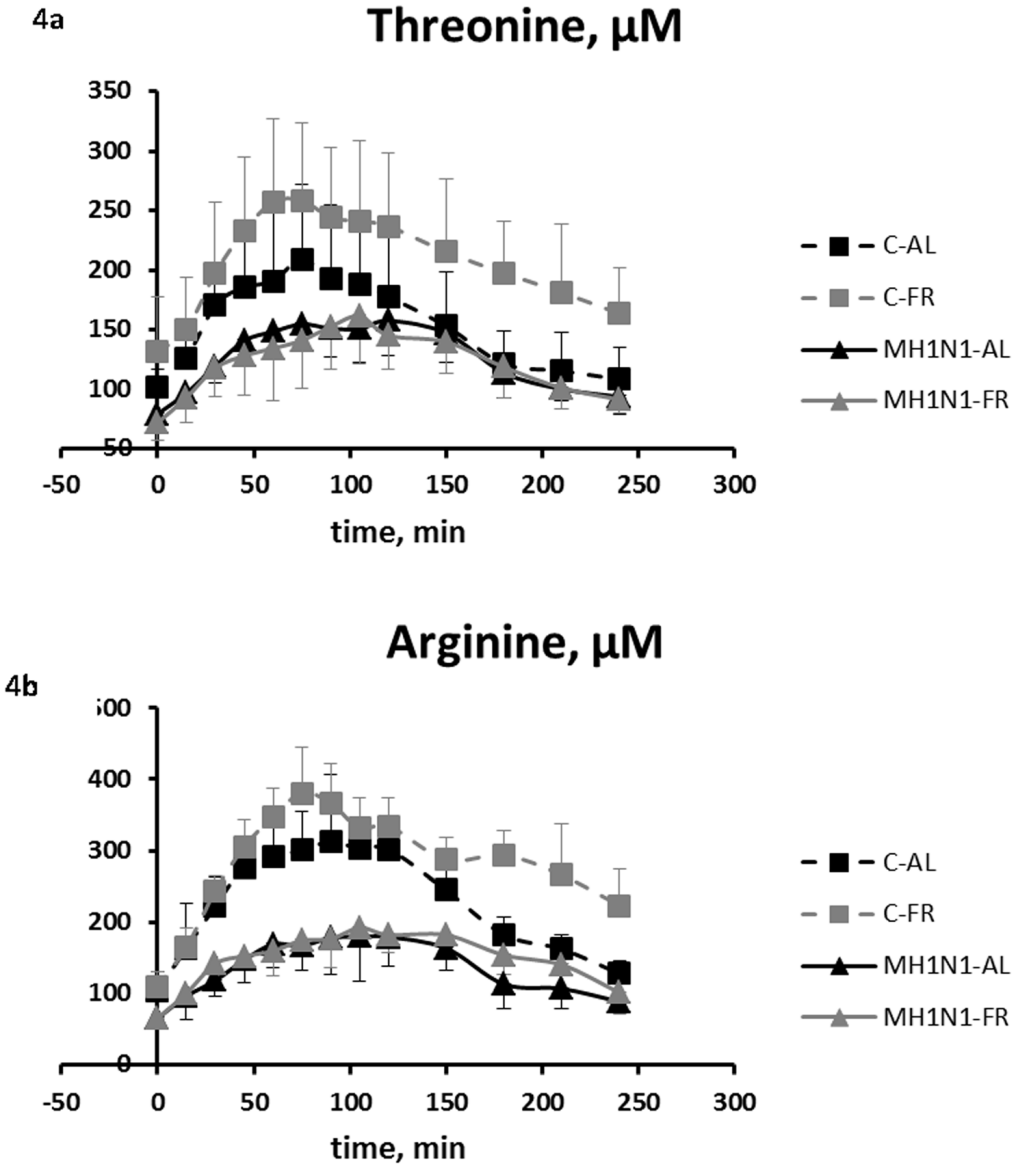
Postprandial kinetics of plasma threonine (3a) and arginine (3b) concentrations (µM) measured in pigs. Average plasma concentrations were measured after an overnight fasting and then during the 4ïc fluid administration in co-infected (MH1N1) and control (C) pigs fed *ad libitum* (AL) or feed restricted (FR): C-AL (black dashed line), C-FR (gray dashed line), MH1N1-AL (black solid line) and MH1N1-FR groups (gray solid line). Values are means +/− SD. P values for threonine: P_I_<0.05 from basal to 240 min; P_FR_<0.05 from 180 to 240 min; P_I×FR_<0.05 from 180 to 240 min. P values for arginine: P_I_<0.01 from basal to 240 min; P_FR_<0.05 from 150 to 240 min; P_I×FR_<0.05 from 180 to 240 min.

All AA concentrations increased after the meal, to reach a peak between 30 min and 60 min, and decreased thereafter, as shown for threonine and arginine in Figure 4 (see Tables S2, S3 and S4 for other AA). Both threonine and arginine exhibited a singular response to co-infection. Indeed, their postprandial plasma concentrations were lower in MH1N1 than in control pigs at any time (P_I_<0.05) and affected to a greater extent than the other AA.

Average plasma AUC calculated during the 4 hours following the meal test, including basal concentrations, are presented in Table 4. The average AUC of threonine, histidine and asparagine were lower in MH1N1 than in C pigs (P_I_<0.05). For AA whose AUC were affected by feeding level only, tryptophan was lower in FR than in AL pigs (P_FR_ = 0.04), whereas leucine, glutamate and ornithine were greater in FR than in AL pigs (P_FR_<0.05). Feeding level and infection had cumulative effects on AUC of arginine, citrulline, and tyrosine: their AUC were lower in MH1N1 than in C groups (P_I_<0.01). Arginine and citrulline AUC were also higher (P_FR_<0.05) in FR than in AL pigs, by contrast to tyrosine AUC that was lower (P_FR_ = 0.03). A significant I×FR interaction (P_I×FR_<0.05) was found for a last group of AA corresponding to lysine, alanine, aspartate, glutamate, ornithine and proline. Aspartate, glutamate and ornithine AUC were greater in FR than in AL pigs (P_FR_<0.003), but the difference was significant only for Control pigs. Alanine, proline and lysine AUC were not affected by co-infection and feed restriction, but feed restriction increased their AUC in Control pigs but not in MH1N1 pigs.

**Table 4:** Average area under the curve for plasma amino acid concentrations (µMxmin) calculated during the 4 hours of blood sampling following the meal test (basal values are included) in control (C) and co-infected (MH1N1) pigs fed *ad libitum* (AL) or feed restricted (FR).

Experimental groups	C-AL	C-FR	MH1N1-AL	MH1N1-FR	SEM	P-value		
n	4	4	6	5		I	FR	I×FR
Essential amino acids								
Arginine	55204	69185	33373	36804	15618	<0.0001	0.006	ns
Histidine	21053	18484	17224	16841	2840	0.04	ns	ns
Isoleucine	34341	38364	33060	35978	5278	ns	ns	ns
Leucine	44772	57708	46112	49919	5580	ns	0.03	ns
Lysine	31900^a^	45580^b^	39524^ab^	36823^ab^	7908	ns	ns	0.02
Methionine	8942	8784	9181	8267	1272	ns	ns	ns
Phenylalanine	30620	29199	33459	30351	4053	ns	ns	ns
Threonine	37026	50320	31280	29766	10911	0.003	ns	ns
Tryptophan	19172	17956	19029	15434	2641	ns	0.04	ns
Valine	70709	79348	72582	75081	8477	ns	ns	ns
Non essential amino acids								
Alanine	113836^a^	154622^b^	159697^b^	146270^ab^	28570	ns	ns	0.03
Aspartate	3035^a^	5292^b^	4967^b^	4648^b^	991	0.03	0.003	0.0003
Asparagine	32019	37980	29517	28969	6374	0.05	ns	ns
Citrulline	20405	27588	18220	19015	4831	0.004	0.03	ns
Glutamine	148005	130536	155686	144816	19914	ns	ns	ns
Glutamate	20849^a^	29699^ab^	35846^b^	33468^b^	7540	ns	0.002	0.04
Glycine	216882	231114	231028	211025	33315	ns	ns	ns
Ornithine	24667^a^	37587^b^	28137^a^	30719^ab^	6111	ns	0.002	0.03
Proline	70889^a^	88489^b^	78087^ab^	75439^ab^	10749	ns	ns	0.04
Serine	40418	39797	37876	39214	4443	ns	ns	ns
Tyrosine	27811	27036	22344	14902	6148	0.0001	0.03	ns

Values are least square means. n = number of pigs. SEM = standard error of the mean; I = Infection; FR = Feed restriction; ns = not significant (P>0.05).

a,b : values with different letters are significantly different with P<0.05; ns = not significant: P>0.05 for I×FR and P>0.1 for I and FR.

## Discussion

In this study, we evaluated the impact of a feed restriction on the ability of pigs to resist and to tolerate a Mhp/H1N1 co-infection. More specifically, we investigated the changes in glucose, urea and AA metabolism caused by the feed restriction and/or the co-infection.

As expected, following H1N1 inoculation, Mhp pre-infected pigs that were fed *ad libitum* developed coughing, hyperthermia, increased respiratory rates and a weight loss due to reduced feed consumption. This was in agreement with the symptoms we observed in similar previous studies [17], [18]. In the present experiment, we showed that feed restriction induced a decrease in rectal temperature that could be caused by a decrease in heat production due to feed digestion [26]. Following H1N1 infection, feed restricted pigs, as compared to pigs fed *ad libitum*, presented a shorter hyperthermia period, did not decrease their feed consumption and did not lose weight. Even if the feed restriction had no impact on pneumonia lesions, on Mhp and H1N1 spreading in lungs and on virus shedding in nasal swabs, these data suggest that a feed restriction would limit the clinical outcomes of flu in Mhp/SIV co-infected pigs and would have a beneficial impact on the zootechnical performance of these animals.

Interestingly, it can be noticed that both the FR and AL groups ate the same quantity of feed (on average 30 g of food per kg of body weight) during the 3 first days following the H1N1 inoculation but the MWG was lower in MH1N1-AL. If a difference in gastrointestinal tract emptying between AL and FR pigs is probably not an explanation, a difference in the mass of visceral tissues could not be ruled out [26]. Otherwise, MH1N1-FR pigs might have developed a lower inflammatory response, as suggested by their shorter hyperthermia. Thus, metabolic disturbances caused by the inflammatory response may have been less pronounced for MH1N1-FR pigs than for MH1N1-AL pigs, which resulted in a higher MWG despite a similar feed intake.

Indeed, the analysis of post-prandial nutrient variations suggests a difference in the utilization of glucose and AA between groups. Postprandial nutrient utilization was investigated after a meal standardized for size, duration and composition. Nutrients whose metabolism was altered by co-infection or feed restriction were those whose profile of appearance and disappearance in the plasma after the meal were altered in comparison to control groups. It appeared that postprandial glycemia profiles were greatly influenced by both feed restriction and co-infection. Generally speaking, gluconeogenesis is not a major metabolic pathway in fed growing pigs [27] since the feed supplies a large amount of carbohydrate (more than 40%) and few lipids (less than 4%). Consequently, the postprandial glucose profile mainly reflects the fate of dietary glucose. Co-infection elicited a lower post-prandial glucose appearance. This clearly reveals that infection leads to changes in glucose metabolism and partitioning between organs. Glucose utilization is enhanced during the inflammatory state to meet the high energy requirements associated with immune activation and inflammatory process. For example, the immune cells require energy for proliferation but the use of glucose as an energy source remains debatable as different types of immune cells could preferentially use glutamine [28]–[30] or glucose [31]. Furthermore, *in vivo* quantitative measurements of glucose fluxes in growing pigs showed that muscle glucose utilization is increased during inflammation, thereby contributing to reduce glycemia [32]. In FR pigs, the glycemia profile indicated that little glucose appeared in the blood and/or that glucose was rapidly removed from the blood. The lower postprandial glucose concentrations during the first hour following the meal may be related to an increased use of glucose for reconstitution of the glycogen pool in both the liver and the muscle [33].

Plasma urea is produced by the liver from the catabolism of AA derived from the absorption of dietary protein as well as the catabolism of body proteins. In our study, plasma urea concentrations increased during the first 4 postprandial hours as previously reported [34], due to the catabolism of dietary amino acids. The lower plasma urea concentration reported in co-infected pigs was unexpected because inflammation is known to enhance muscle protein catabolism and urea excretion [35]. The spontaneous reduced feed intake observed during the first 3 days after the inoculation of H1N1 virus in AL pigs could have prevented the increase in urea synthesis that is mainly influenced by protein intake [34]. Alternatively, AA may have been metabolized by the liver for the synthesis of acute phase proteins [36], thus preserving amino acids from catabolism. In the present study, inflammation was confirmed in MH1N1-FR and MH1N1-AL groups by measuring an increase in plasma haptoglobin concentrations after H1N1 inoculation, with same levels in both infected groups (data not shown), as similarly observed in a previous study [18].

Not all AA profiles were affected by co-infection suggesting a specific impact of infection on some AA rather than a global effect on protein metabolism. As in co-infected pigs in our study, lower tyrosine plasma concentrations were reported in pigs suffering from clinical dysentery and could be related to the incorporation of this AA into acute phase proteins [37]. Among essential AA, plasma concentrations of histidine, arginine and threonine were also reduced in co-infected pigs. Lower histidine concentrations were reported in weaned pigs infected with *Escherichia coli* but this effect was nonspecific since many other AA showed a similar response [38]. The involvement of arginine in the immune and inflammatory processes is fully documented [1]. Arginine is converted into nitric oxide (NO) by the action of inducible NO synthase (iNOS), of which the activity is enhanced by influenza virus infection [39]. Some strains of *Mycoplasma* are also known to activate iNOS [40]. Moreover, cells infected with influenza virus use arginine to synthesize an arginine rich component [41]. Arginine, with glutamate, is also the precursor of citrulline synthesis in the gut [42]. Lower arginine concentrations may also limit the synthesis of citrulline, for which the concentrations were reduced in MH1N1 compared to C pigs, contributing to glutamate accumulation in the plasma. The plasma arginine profile, like that of ornithine and citrulline, was affected by feed restriction. The metabolism of these three AA is strongly interconnected in the urea cycle that takes place in the liver, since both ornithine and citrulline are synthesized from arginine. Their greater postprandial concentrations in FR pigs could be related to urea synthesis, although the lack of effect of feed restriction on the urea profile did not corroborate this hypothesis.

Threonine is abundant in glycoproteins such as intestinal and tracheal mucins [43], [44] and immunoglobulins [45]. In healthy pigs, the intestinal tissues extract dietary threonine in great amounts [46] and gut inflammation increases intestinal threonine uptake and mucin synthesis [47] thereby reducing threonine availability for the other tissues. In our experiment, it seems unlikely that the reduced appearance of threonine observed in the plasma during the postprandial period was due to an increased use by the gastrointestinal tract. Indeed, *Mycoplasma* and H1N1 target the respiratory rather than the gastrointestinal tract [14], [15]. Both pathogens enhance mucin secretion in the respiratory tract [48], [49], but no evidence that threonine is also abundant in mucins of the porcine respiratory tract has been reported yet. Finally, threonine could be used for the increased production of total immunoglobulins that was demonstrated in co-infected groups. In growing pigs, the adequate threonine dietary supply required for production of immunoglobulins may be higher than that required for growth [50].

Interestingly, co-infection differently affected lysine, alanine, aspartate, glutamate, proline and ornithine if the pigs were fed ad libitum or feed restricted. In our experiment, both feed restriction and co-infection impaired growth rate and would help to explain the accumulation in the plasma of lysine, an essential AA mainly used for protein synthesis. In co-infected pigs, the absence of a cumulative effect of feed restriction on the plasma concentrations of those AA may be explained by the greater mean daily gain after H1N1 inoculation, and thus by the greater protein deposition, of MH1N1-FR pigs compared with MH1N1-AL pigs. Alternatively, these AA may have been used in metabolic pathways related to immune response and contributing to energy supply for alanine [51], to nitrogen recycling and removal for glutamate, aspartate, proline and ornithine and to the synthesis of immune proteins for lysine [52]. Whether or not this could have contributed to the positive effect of feed restriction on the growth of infected pigs remains speculative.

In conclusion, our study showed a beneficial, even if limited, effect of feed restriction on the outcomes of H1N1 infection in pigs pre-infected with Mhp. We showed, for the first time, that both co-infection and feed restriction modified the postprandial kinetics of glucose and AA concentrations, revealing major changes in nutrient metabolism. Further studies will be necessary to investigate the role of AA such as threonine and arginine during co-infection and to find out if a supplemented feed would improve the resistance and the tolerance of the pig to the disease. For instance, the correlation between the magnitude of clinical responses and metabolic responses deserve to be studied on larger experimental groups. Nevertheless, these first results already indicate that feeding practices could provide a means to prepare animals to overcome an influenza infection, especially in the context of the Porcine Respiratory Disease Complex.

## Supporting Information

Table S1 **Composition of the feed (as fed basis).** * Detailed supplementation not disclosed by the manufacturer. Contained acidifiers, vitamins and minerals, enzymes and linseed oil.
(DOC)

Table S2 **Average basal plasma amino acid concentrations (µM) measured after an overnight fasting in control (C) and co-infected (MH1N1) pigs fed** ***ad libitum*** **(AL) or feed restricted (FR).** Values are least square means. n = number of pigs. SEM = standard error of the mean; I = Infection; FR = Feed restriction; ns = not significant: P>0.05 for I×FR and P>0.1 for I and FR. * Statistical analysis was performed on log-transformed values. Values are 4.65, 4.72, 7.70, and 4.71 for C-AL, C-FR, MH1N1-AL, and MH1N1-FR respectively.
(DOC)

Table S3 **Average plasma amino acid concentrations (µM) measured 60 minutes after the distribution of the meal test in control (C) and co-infected (MH1N1) pigs fed** ***ad libitum*** **(AL) or feed restricted (FR).** Values are least square means. n = number of pigs. SEM = standard error of the mean; I = Infection; FR = Feed restriction; ns = not significant (P>0.05). ^a^,^b^: values with different letters are significantly different with P<0.05.; ns = not significant: P>0.05 for I×FR and P>0.1 for I and FR. * Statistical analysis was performed on log-transformed values. Values are 2.10, 2.34, 1.94, and 1.99 for C-AL, C-FR, MH1N1-AL, and MH1N1-FR respectively.
(DOC)

Table S4 **Average plasma amino acid concentrations (µM) measured 240 minutes after the distribution of the meal test in control (C) and co-infected (MH1N1) pigs fed** ***ad libitum*** **(AL) or feed restricted (FR).** Values are least square means. n = number of pigs. SEM = standard error of the mean; I = Infection; FR = Feed restriction; ns = not significant (P>0.05). ^a^,^b^: values with different letters are significantly different with P<0.05.; ns = not significant: P>0.05 for I×FR and P>0.1 for I and FR. * Statistical analysis was performed on log-transformed values. Values for threonine are 2.02, 2.20, 1.97 and 1.96 and values for alanine are 2.50, 2.63, 2.60 and 2.64, for C-AL, C-FR, MH1N1-AL, and MH1N1-FR, respectively.
(DOC)
