# Supplementary material for: Effect of Feed Restriction on Performance and Postprandial Nutrient Metabolism in Pigs Co-Infected with Mycoplasma hyopneumoniae and Swine Influenza Virus
Source: PLoS One. 2014 Aug 7;9(8):e104605. doi: 10.1371/journal.pone.0104605 (PMC4125196; doi:10.1371/journal.pone.0104605)
Supplement: Table S1 — Composition of the feed (as fed basis). * Detailed supplementation not disclosed by the manufacturer. Contained acidifiers, vitamins and minerals, enzymes and linseed oil. (DOC) [file pone.0104605.s001.doc]

**Table S1**: Composition of the feed (as fed basis)

|  | Starter | Growing |
| --- | --- | --- |
| Ingredients, g/kg |  |  |
| Corn | 200 | 150 |
| Wheat | 100 | 100 |
| Barley | 372 | 310 |
| Oat | - | 40 |
| Wheat bran | 50 | 75 |
| Soyabean meal | 200 | 152 |
| Pea | 25 | 50 |
| Alfalfa | - | 50 |
| Calcium carbonate | - | 2 |
| Bicalcium phosphate | 12.3 | 13.2 |
| Clay | 10 | 10 |
| Salt | 3.5 | 3.5 |
| L-lysine HCl | 2.2 | 1 |
| DL-methionine | 0.5 | - |
| L-threonine | 0.5 | - |
| Feed additives* | 24 | 43.3 |
| Chemical composition, % |  |  |
| Dry matter, % | 87.25 | 87.52 |
| Minerals % | 6.72 | 7.06 |
| Crude Protein,% | 16.76 | 16.01 |
| Crude fat | 2.18 | 2.7 |
| Crude fiber | 4.43 | 6.10 |
| Starch | 46.09 | 42.51 |
| Nutritional composition |  |  |
| Net energy, kJ/kg | 9329 | 8885 |
| Digestible Lysine, % | 0.90 | 0.75 |
| Digestible Methionine, % | 0.28 | 0.22 |
| Digestible Threonine, % | 0.56 | 0.48 |
| Digestible Tryptophane,% | 0.18 | 0.17 |
| Digestible Phosphorus | 0.31 | 0.34 |
| Calcium | 0.97 | 1.05 |
