# Supplementary material for: Effect of Feed Restriction on Performance and Postprandial Nutrient Metabolism in Pigs Co-Infected with Mycoplasma hyopneumoniae and Swine Influenza Virus
Source: PLoS One. 2014 Aug 7;9(8):e104605. doi: 10.1371/journal.pone.0104605 (PMC4125196; doi:10.1371/journal.pone.0104605)
Supplement: Table S2 — Average basal plasma amino acid concentrations (µM) measured after an overnight fasting in control (C) and co-infected (MH1N1) pigs fed ad libitum (AL) or feed restricted (FR). Values are least square means. n = number of pigs. SEM = standard error of the mean; I = Infection; FR = Feed restriction; ns = not significant: P>0.05 for I×FR and P>0.1 for I and FR. * Statistical analysis was performed on log-transformed values. Values are 4.65, 4.72, 7.70, and 4.71 for C-AL, C-FR, MH1N1-AL, and MH1N1-FR respectively. (DOC) [file pone.0104605.s002.doc]

**Table S2: Average basal plasma amino acid concentrations (µM) measured after an overnight fasting in control (C) and co-infected (MH1N1) pigs fed *ad libitum* (AL) or feed restricted (FR).**

| Experimental groups | C-AL | C-FR | MH1N1-AL | MH1N1-FR | SEM |  | | |
| --- | --- | --- | --- | --- | --- | --- | --- | --- |
|  |  |  |  |  |  | P-value | | |
| n | 4 | 4 | 6 | 5 |  |  |  |  |
|  |  |  |  |  |  | I | FR | I x FR |
| Essential amino acids | | | | | | | | |
| Arginine | 104 | 110 | 65 | 65 | 26.7 | 0.0001 | ns | ns |
| Histidine | 56 | 40 | 40 | 35 | 10.4 | 0.01 | 0.008 | ns |
| Isoleucine | 106 | 106 | 103 | 104 | 18.3 | ns | ns | ns |
| Leucine* | 144 | 142 | 152 | 145 | 21.6 | ns | 0.03 | ns |
| Lysine | 55 | 61 | 75 | 63 | 26.8 | ns | ns | ns |
| Methionine | 30 | 27 | 29 | 25 | 4.4 | ns | 0.07 | ns |
| Phenylalanine | 72 | 60 | 77 | 67 | 12.0 | ns | 0.04 | ns |
| Threonine | 102 | 131 | 79 | 72 | 32.2 | 0.003 | ns | ns |
| Tryptophan | 53 | 46 | 52 | 41 | 7.4 | ns | 0.004 | ns |
| Valine | 237 | 233 | 239 | 232 | 30.4 | ns | ns | ns |
| Non essential amino acids | | | | | | | | |
| Alanine | 339 | 465 | 429 | 488 | 82.1 | 0.09 | 0.009 | ns |
| Aspartate | 11 | 15 | 15 | 15 | 2.8 | 0.08 | ns | ns |
| Asparagine | 60 | 62 | 55 | 54 | 9.6 | ns | ns | ns |
| Citrulline | 66 | 73 | 60 | 59 | 9.4 | 0.02 | ns | ns |
| Glutamine | 504 | 432 | 483 | 492 | 62.9 | ns | ns | ns |
| Glutamate | 96 | 119 | 140 | 133 | 23.7 | 0.005 | ns | ns |
| Glycine | 965 | 1013 | 963 | 869 | 133.3 | ns | ns | ns |
| Ornithine | 53 | 78 | 56 | 64 | 12.9 | ns | 0.002 | ns |
| Proline | 178 | 204 | 193 | 197 | 15.1 | ns | 0.03 | ns |
| Serine | 121 | 119 | 127 | 120 | 14.2 | ns | ns | ns |
| Tyrosine | 67 | 49 | 51 | 33 | 15.6 | 0.006 | 0.003 | ns |
