# Supplementary material for: Effect of Feed Restriction on Performance and Postprandial Nutrient Metabolism in Pigs Co-Infected with Mycoplasma hyopneumoniae and Swine Influenza Virus
Source: PLoS One. 2014 Aug 7;9(8):e104605. doi: 10.1371/journal.pone.0104605 (PMC4125196; doi:10.1371/journal.pone.0104605)
Supplement: Table S3 — Average plasma amino acid concentrations (µM) measured 60 minutes after the distribution of the meal test in control (C) and co-infected (MH1N1) pigs fed ad libitum (AL) or feed restricted (FR). Values are least square means. n = number of pigs. SEM = standard error of the mean; I = Infection; FR = Feed restriction; ns = not significant (P>0.05). a,b: values with different letters are significantly different with P<0.05.; ns = not significant: P>0.05 for I×FR and P>0.1 for I and FR. * Statistical analysis was performed on log-transformed values. Values are 2.10, 2.34, 1.94, and 1.99 for C-AL, C-FR, MH1N1-AL, and MH1N1-FR respectively. (DOC) [file pone.0104605.s003.doc]

**Table S3: Average plasma amino acid concentrations (µM) measured 60 minutes after the distribution of the meal test in control (C) and co-infected (MH1N1) pigs fed *ad libitum* (AL) or feed restricted (FR).**

| Experimental groups | C-AL | C-FR | MH1N1-AL | MH1N1-FR | SEM |  | | |
| --- | --- | --- | --- | --- | --- | --- | --- | --- |
|  |  |  |  |  |  | P-value | | |
| n | 4 | 4 | 6 | 5 |  |  |  |  |
|  |  |  |  |  |  | I | FR | I x FR |
| Essential amino acids | | | | | | | | |
| Arginine* | 291^a^ | 347^b^ | 169^a^ | 159^a^ | 87.4 | <0.0001 | 0.003 | 0.03 |
| Histidine | 105 | 95 | 79 | 71 | 20.7 | 0.007 | ns | ns |
| Isoleucine | 205 | 182 | 168 | 164 | 30.9 | 0.06 | ns | ns |
| Leucine | 250 | 291 | 242 | 233 | 36.7 | 0.05 | ns | ns |
| Lysine | 204^ab^ | 267^b^ | 215^ab^ | 185^a^ | 46.0 | 0.06 | ns | 0.02 |
| Methionine | 49 | 54 | 46 | 39 | 10.4 | 0.08 | ns | ns |
| Phenylalanine | 148 | 146 | 145 | 126 | 22.7 | ns | ns | ns |
| Threonine | 191 | 257 | 148 | 134 | 64.0 | 0.002 | ns | ns |
| Tryptophan | 93 | 88 | 88 | 71 | 16.0 | ns | ns | ns |
| Valine | 364 | 400 | 354 | 335 | 47.4 | 0.09 | ns | ns |
| Non-essential amino acids | | | | | | | | |
| Alanine | 675^a^ | 872^b^ | 806^ab^ | 723^ab^ | 134.2 | ns | ns | 0.03 |
| Aspartate | 17^a^ | 25^b^ | 21^ab^ | 19^ab^ | 4.3 | ns | ns | 0.006 |
| Asparagine | 177 | 215 | 141 | 130 | 49.5 | 0.05 | ns | ns |
| Citrulline | 88 | 112 | 74 | 74 | 20.8 | 0.002 | ns | ns |
| Glutamine | 719 | 616 | 706 | 612 | 103.9 | ns | 0.05 | ns |
| Glutamate | 107 | 159 | 176 | 169 | 39.4 | 0.02 | ns | ns |
| Glycine | 1031 | 1133 | 1044 | 907 | 182.2 | ns | ns | ns |
| Ornithine | 115 | 151 | 112 | 116 | 22.0 | 0.02 | 0.02 | ns |
| Proline | 362^ab^ | 456^b^ | 350^a^ | 332^a^ | 68.1 | 0.02 | ns | 0.04 |
| Serine | 205 | 216 | 187 | 174 | 32.6 | 0.05 | ns | ns |
| Tyrosine | 130^b^ | 131^b^ | 101^b^ | 64^a^ | 31.3 | <0.0001 | 0.04 | 0.03 |
