# Supplementary material for: Effect of Feed Restriction on Performance and Postprandial Nutrient Metabolism in Pigs Co-Infected with Mycoplasma hyopneumoniae and Swine Influenza Virus
Source: PLoS One. 2014 Aug 7;9(8):e104605. doi: 10.1371/journal.pone.0104605 (PMC4125196; doi:10.1371/journal.pone.0104605)
Supplement: Table S4 — Average plasma amino acid concentrations (µM) measured 240 minutes after the distribution of the meal test in control (C) and co-infected (MH1N1) pigs fed ad libitum (AL) or feed restricted (FR). Values are least square means. n = number of pigs. SEM = standard error of the mean; I = Infection; FR = Feed restriction; ns = not significant (P>0.05). a,b: values with different letters are significantly different with P<0.05.; ns = not significant: P>0.05 for I×FR and P>0.1 for I and FR. * Statistical analysis was performed on log-transformed values. Values for threonine are 2.02, 2.20, 1.97 and 1.96 and values for alanine are 2.50, 2.63, 2.60 and 2.64, for C-AL, C-FR, MH1N1-AL, and MH1N1-FR, respectively. (DOC) [file pone.0104605.s004.doc]

**Table S4: Average plasma amino acid concentrations (µM) measured 240 minutes after the distribution of the meal test in control (C) and co-infected (MH1N1) pigs fed *ad libitum* (AL) or feed restricted (FR).**

| Experimental groups | C-AL | C-FR | MH1N1-AL | MH1N1-FR | SEM |  | | |
| --- | --- | --- | --- | --- | --- | --- | --- | --- |
|  |  |  |  |  |  | P-value | | |
| n | 4 | 4 | 6 | 5 |  |  |  |  |
|  |  |  |  |  |  | I | FR | I x FR |
| Essential amino acids | | | | | | | | |
| Arginine | 128^a^ | 223^b^ | 88^a^ | 101^a^ | 58.6 | <0.0001 | 0.001 | 0.008 |
| Histidine | 62 | 60 | 53 | 58 | 8.8 | ns | ns | ns |
| Isoleucine | 103.4 | 112.8 | 96.7 | 113.7 | 16.9 | ns | ns | ns |
| Leucine | 128 | 148 | 127 | 151 | 20.5 | ns | 0.02 | ns |
| Lysine | 53 | 97 | 78 | 83 | 26.3 | ns | 0.04 | ns |
| Methionine | 27 | 21 | 27 | 24 | 4.8 | ns | 0.03 | ns |
| Phenylalanine | 92 | 95 | 113 | 103 | 14.6 | 0.03 | ns | ns |
| Threonine* | 108^a^ | 164^b^ | 94^a^ | 91^a^ | 35.8 | 0.007 | 0.03 | 0.02 |
| Tryptophan | 58 | 55 | 67 | 48 | 11.6 | ns | 0.03 | ns |
| Valine | 222 | 256 | 225 | 250 | 27 | ns | 0.02 | ns |
| Non-essential amino acids | | | | | | | | |
| Alanine* | 303 | 431 | 442 | 437 | 103.2 | 0.08 | 0.06 | ns |
| Aspartate | 9^a^ | 15^ab^ | 16^b^ | 18^b^ | 4.2 | ns | ns | 0.006 |
| Asparagine | 76 | 108 | 80 | 78 | 20.8 | ns | ns | ns |
| Citrulline | 73^a^ | 112^b^ | 70^a^ | 76^a^ | 19.9 | 0.004 | 0.001 | 0.01 |
| Glutamine | 506 | 473 | 508 | 508 | 62.4 | ns | ns | ns |
| Glutamate | 55 | 74 | 99 | 103 | 25.9 | 0.0008 | ns | ns |
| Glycine | 796 | 824 | 808 | 752 | 122.7 | ns | ns | ns |
| Ornithine | 65^a^ | 145^c^ | 95^ab^ | 109^bc^ | 32.4 | ns | 0.0001 | 0.003 |
| Proline | 217^a^ | 291^b^ | 239^a^ | 238^a^ | 41.4 | ns | 0.04 | 0.04 |
| Serine | 112 | 122 | 118 | 137 | 17.8 | ns | 0.07 | ns |
| Tyrosine | 96^b^ | 87^b^ | 75^ab^ | 54^a^ | 19.3 | 0.0003 | ns | 0.01 |
